# Infection routes, invasion mechanisms, and drug inhibition pathways of human coronaviruses on the nervous system

**DOI:** 10.3389/fnins.2023.1169740

**Published:** 2023-04-17

**Authors:** Ailong Sha, Hongrun Chen

**Affiliations:** ^1^School of Teacher Education, Chongqing Three Gorges University, Chongqing, China; ^2^School of Biology and Food Engineering, Chongqing Three Gorges University, Chongqing, China

**Keywords:** coronavirus, central nervous system, peripheral nervous system, prevention and treatment, drug

## Abstract

So far, numerous studies have reported on how coronaviruses affect the human nervous system. However, these studies mainly focused on the impact of a single coronavirus on the nervous system, and failed to fully report the invasion mechanisms and the rules of symptoms of the seven human coronaviruses. This research can assist medical professionals in identifying the regularity of coronavirus invasion into the nervous system by examining the impacts of human coronaviruses on the nervous system. Meanwhile, the discovery also helps humans to prevent the damage to the human nervous system caused by the more novel coronavirus in advance, thus reducing the rate of disease transmission and fatality caused by such viruses. In addition to describing the structures, routes of infection, and symptomatic manifestations of human coronaviruses, this review also finds that the structures of human coronaviruses correlate with virulence, pathways of infection, and blocking mechanisms of drugs. This review can provide a theoretical basis for the research and development of related drugs, promote the prevention and treatment of coronavirus infectious diseases, and contribute to global epidemic prevention.

## 1. Introduction

Human coronaviruses refer to single-stranded RNA viruses with a protein envelope, including Severe Acute Respiratory syndrome virus (SARS-CoV), Middle East Respiratory Syndrome virus (MERS-CoV), HCoV-229E, HCoV-OC43, HCoV-NL63, and HCoV-HKU1. Many researchers regard these coronaviruses as potent respiratory viruses, ignoring their ability to invade the nervous system (NS). The emergence of a new coronavirus, SARS-CoV-2, which also belongs to the human coronavirus, is brought about by the novel coronavirus disease (COVID-19). Numerous studies have elaborated on the mechanisms, pathways, and scopes of SARS-CoV-2 infestation in humans, and found that it can cause not only intestinal and respiratory diseases in humans (Huang et al., 2020), but also different degrees of damage to the nervous system. It is speculated that other coronaviruses have similar mechanisms of action.

The seven human coronaviruses are classified as beta coronaviruses or alpha coronaviruses. Among them, SARS-CoV-2, MERS-CoV, HCoV-HKU1, HCoV-OC43, and SARS-CoV belong to the branch of beta coronaviruses. Genome analysis showed that SARS-CoV-2 and SARS-CoV had a highly homologic sequence (Huang et al., 2020). Furthermore, the researchers discovered that the two coronaviruses shared a common receptor, the human angiotensin-converting enzyme (ACE2), which was located on the surface of neurons, astrocytes, olfactory epithelial cells, and other cells. SARS-CoV-2 can invade the human central nervous system (CNS) by binding the ACE2 receptors. Comparatively speaking, SARS-CoV and HCoV-NL63 also use ACE2 as receptors, but their binding affinity for the receptor binding domain (RBD) of the S protein is lower than that of SARS-CoV-2 (Lan et al., 2022). After coronaviruses infect the host cells with the help of receptors, microglia cells are activated and release inflammatory factors, such as interleukin-8 (IL-8), triggering a robust immune response that leads to inflammation in the body. A clinical case has confirmed the presence of a large amount of IL-8 in the cerebrospinal fluid (CSF) of some patients with coronavirus-induced encephalitis (Li et al., 2016). It has been speculated that glial cells in the brain can be transformed into aggressive effector cells that participate in specific immune responses, leading to neuronal damage (Nakajima et al., 2001). Additionally, the patients with COVID-19 and HCoV-OC43 had viral RNA in the CSF (Yeh et al., 2004; Moriguchi et al., 2020). The results of an MRI showed that some MERS patients had lesions in the white matter of the temporal lobes and parietal lobes, subcortical regions, basal ganglia, corpus callosum, pons, cerebellum, and upper cervical medulla, as well as an intracerebroventricular expansion (Arabi et al., 2015). These cases illustrated that the coronavirus could damage and destroy the internal structure of the CNS. Moreover, some patients infected with coronaviruses (e.g., SARS-CoV-2 and SARS-CoV) have also presented with neuromuscular symptoms (Vonck et al., 2020), taste loss (Martinez et al., 2020), etc. These manifestations were due to the coronavirus infection of the peripheral nervous system (PNS), which innervated the sensory and motor nerves. Nevertheless, alpha coronaviruses like HCoV-229E and HCoV-NL63 were hardly ever discussed in the NS field, and it was once thought that they could not influence the NS. It is thus clear that previous studies have failed to elucidate the effects of all coronaviruses on the NS comprehensively.

Therefore, this paper will describe the influence of the above seven coronaviruses on the NS, summarize and analyze the mechanisms of action and symptoms of these coronaviruses attacking the NS, as well as the connection between these coronaviruses and disease prevention and treatment. By exploring the influence of different coronaviruses on the human nervous system, the regularity of coronaviruses’ invasion into the nervous system of the body can be found. This discovery can provide a reference for the prevention and control of the more novel coronaviruses that may appear in the future. Meanwhile, it also can help humans to prevent the damage to the human nervous system caused by the more novel coronavirus in advance, thus reducing the rate of transmission and fatality of disease caused by such viruses, and contributing to global epidemic prevention and control.

## 2. Structural composition, function, and blocking means of coronavirus proteins

Coronaviruses are named because of their coronal structure under electron microscopy (Rabaan et al., 2020). The positive-stranded RNA of this virus serves as the genomic material, while the outer envelope bears glycoprotein spikes. Coronavirus RNA replicates in the cytoplasm of the host cell. This protein is encoded by the coronavirus genome and is mainly divided into five structural proteins: spike (S), envelope (E), membrane (M), nucleocapsid (N), and hemagglutinin-esterase (HE) proteins. The SARS-CoV-2, SARS-CoV, HCoV-229E, and HCoV-NL63 genomes have four genes that encode S, M, N, and E structural proteins, respectively. HCoV-OC43 and HCoV-HKU1 have a different gene that expresses the HE protein (Rabaan et al., 2020). Among the above structural proteins, the S protein, the HE protein, the E protein, and the N protein have a significant impact on the human nervous system.

### 2.1. Spike protein

The S protein is a critical mediator of coronavirus infection of the host cell, like SARS-CoV-2, SARS-CoV, MERS-CoV, and HCoV-NL63 require binding to the receptor with the help of the RBD on the S protein. Additionally, the combining capacity of RBD is used to judge the virulence of coronavirus, and the stronger the combining capacity, the greater the virulence. For example, the binding of the S protein to ACE2 relies on the cleavage by the host cell proteases at the S1/S2 cleavage sites, and the process requires the assistance of transmembrane serine protease (TMPRSS2) (Hoffmann et al., 2020a; Matsuyama et al., 2020). The S1 subunit with RBD can bind to the surface receptors of the host cell, and help the coronavirus attach to cells. The S2 subunit mediates the fusions of coronavirus-cell and cell-cell membranes, and facilitates coronavirus entry into the host cells.

When coronaviruses infect cells, there are two ways to enter the cell with the help of the S protein: Firstly, after the S1 protein binds to the receptor, the coronavirus uses TMPRSS2 on the host cell to cleave the S protein, and its outer membrane fuses with the host cell membrane, thereby infecting the host cell. Secondly, the coronavirus enters the host cell directly with the receptor through endocytosis. The above processes are inseparable from the S protein. The ability of the coronavirus to combine with host cells will be severely compromised if the S protein is inactivated, which lowers the infectiousness and virulence of the coronavirus. The S protein hence can be regarded as a crucial indicator of the virulence of coronavirus. Therapy of the coronavirus can be achieved by disrupting the structure and activity of such proteins. However, it has been found that TMPRSS2 can be inhibited by protease inhibitors, thus reducing the infectivity of coronaviruses. Camostat is the first serine protease inhibitor proven to inhibit TMPRSS2 (Hoffmann et al., 2020a). Soon after, more TMPRSS2 inhibitors appeared, including nafamostat mesylate (Hoffmann et al., 2020b; Yamamoto et al., 2020) and analogs of arbidol (Choudhary and Silakari, 2020), which were shown to interfere with the entry of coronaviruses (such as SARS-CoV-2) into host cells.

Lun et al. (2021) found that the expression of membrane E3 ubiquitin ligases (MARCH 8) could degrade the S protein of SARS-CoV-2, thus affecting the coronavirus infection. Accordingly, the S protein can be utilized in the research and development of coronavirus treatment drugs. Besides, for treating coronavirus-related diseases, it may be considered whether there is a substance that can bind to the S protein without significant harm to the human body. A new antibody, single-domain antibodies (VHHs), was reported to be extracted from the blood of alpaca Winters, which bound to the S protein on the surface of SARS-CoV-2 and “neutralized” the potential hazard (Wrapp et al., 2020). Two other antibodies that could neutralize SARS-CoV and MERS-CoV were also found in winter’s blood, suggesting that this study had important implications for treating coronaviruses.

### 2.2. Hemagglutinin-esterase (HE), envelope (E), and nucleocapsid (N) proteins

The HE protein can bind to sialic acid on the surface glycoproteins of coronavirus particles (e.g., HCoV-OC43, HCoV-HKU1) (Lang et al., 2020), and it can use this binding and the esterase activity to facilitate S protein-mediated coronavirus entry into the host cell and exacerbate coronavirus transmission in the mucosa (Rabaan et al., 2020). One study suggested that the lack of fully active HE protein would lead to decreased diffusivity and neurotoxicity of HCoV-OC43 in the NS (Dubé et al., 2018), so the activity of HE protein could be considered as one of the indicators for judging the toxicity of coronavirus.

The E protein has ion channel activity and is a “target” for calcium ions. Under the action of calcium ions, the E protein stimulates the inflammasome, prompts the inflammasome to secrete inflammatory factors, enhances the inflammatory response, and finally destroys the NS (Nieto-Torres et al., 2015). Moreover, SARS-CoV overstimulates the NF-κB inflammatory pathway due to the presence of E protein (DeDiego et al., 2014), and interacts with the cellular protein syntenin through its PDZ binding element to trigger the activation of p38 MAPK (Jimenez-Guardeño et al., 2014). These signaling cascades lead to increased inflammation and immunopathology, and damage to the neurons and the internal structure of the NS. The N protein is also an essential structural protein that can encapsulate viral RNA, and play a key role in viral replication, transcription, and genome assembly together with NSPs (non-structural proteases) (Abdel-Moneim et al., 2021).

After learning the destruction mechanism of the above coronavirus structural proteins in the human body, people can reduce the transmission rate of coronaviruses by disrupting or binding the chemical structure of these structural proteins with drugs. Therefore, before the coronavirus invades the body, spraying 75% of the volume fraction of alcohol can destroy the structure and properties of the coronavirus protein, and achieve the effect of eliminating the coronavirus.

## 3. Invasive mechanisms

The NS includes the CNS and the PNS. The CNS controls brain movement and the body’s homeostasis, while the PNS is closely related to the sensations and movements of the body and internal organs. When the coronavirus enters the body and attacks the NS, it is more susceptible because the peripheral nerves (PNs) are directly connected to the local tissues. Further, the coronavirus can cross the blood-brain barrier (abbreviated as BBB, which includes pericytes, endothelial cells, astrocytes, and basement membrane) to reach and destroy the CNS. The primary function of BBB is to maintain a relatively constant brain environment and protect the brain tissue and the CSF. However, many studies have found coronavirus particles in human cerebrospinal fluid, suggesting that coronaviruses can destroy and cross the BBB. The following section is a comprehensive description of the main pathways and mechanisms of the CNS and PNS damage caused by coronaviruses.

### 3.1. Mechanisms of the CNS injury caused by coronaviruses

The CNS consists of the brain and spinal cord, the central part of the NS. The brain is composed of the brain stem, cerebellum, etc., and there are glossopharyngeal nerve and vagus nerve on the side of the medulla oblongata in the brain stem. The cerebellum receives information from the brain stem, spinal cord, and cerebral cortex, and communicates with all three through the feedback loops. This process can not only coordinate the voluntary movement initiated by the cerebral cortex, but also regulate the activities of motor neurons in the brain stem and the anterior horn of the spinal cord, thus coordinating the contraction activity and muscle tension of all muscle groups in the body. Correlatively, the spinal cord includes gray matter and white matter. The gray matter is the site of an extensive collection of the nerve cell body. It innervates skeletal muscle, smooth muscle, cardiac muscle, and glands. The white matter consists of nerve fibers that can carry afferent impulses from the spinal cord to the brain, or efferent impulses from the brain to the spinal cord to form various conduction tracts, such as the corticospinal tract. Because the operation of the aforementioned human physiological structure can affect human behavior and state, clinical patients will exhibit related movement changes and mental states due to the NS damage. Kaur et al. (2020) found that a SARS-CoV-2 patient had decreased sensation in the limbs, which later deteriorated into flaccid quadriplegia and respiratory failure. After ruling out other viral infections, a nasopharyngeal swab of the patient revealed a positive test. In addition, a study described the clinical manifestations of two patients infected with HCoV-OC43 (Sharma et al., 2019). One patient developed unilateral facial nerve palsy with progressive lower limb weakness, which might be related to damage to the trigeminal nerve or pons. Another patient presented with medulla oblongata paralysis, which manifested as dysphagia and respiratory distress, which may have originated from brainstem injury. Helms et al. (2020) observed that 39 out of 58 patients with severe COVID-19 in Strasbourg, France, showed signs of the corticospinal tract, manifested as limb weakness and decreased ability of voluntary movement, which was associated with medulla oblongata infection.

Therefore, a preliminary diagnosis of the link between the coronavirus and the CNS can be made based on patient symptoms. The cellular condition of damaged tissue needs to be detected by the CSF or other brain tissue material, or by electron microscopy, which can further confirm the mode of the coronavirus invasion of the CNS. Recently, the symptoms (such as encephalopathy, encephalitis/meningitis, anosmia, and neurovascular disease) and testing (presence of COVID-19 RNA in the CSF) of the COVID-19 patient also support this assertion. Numerous investigations have also demonstrated that diverse coronavirus types penetrate the central nervous system (CNS) via multiple paths, triggering various neurological disorders. The characteristics of coronavirus invasion into the CNS can be summarized as four pathways: olfactory system pathway, receptor binding infection of cerebral vascular cells, blood circulation infection of mononuclear/macrophages (Trojan horse mechanism), and neurons retrograde across synapses.

#### 3.1.1. Olfactory system pathway

The olfactory system pathway mainly involves the transmission and destruction of the CNS by a coronavirus from olfactory epithelial cells through the olfactory nerve and the olfactory bulb. Studies have shown that after the administration of drugs in the nasopharynx of mice, the fluorescent-labeled drugs formed a transmission route. This pathway travelled from the nasopharynx to the olfactory bulb, its leading edge, the interior of the olfactory bulb and tissues, and eventually the central nervous system (Yin et al., 2010). Therefore, it can be inferred that the olfactory system pathway was one of the vital pathways to the CNS. ACE2 receptor has been found in the olfactory epithelial cells. SARS-CoV-2 and SARS-CoV can damage the olfactory epithelial cells by binding to ACE2 receptor at this site. The response will stimulate the immune response in the body, damage the normal working olfactory nerve, and then affect the structure and function of the CNS, and hinder the “NS work.” The TMPRSS2 can also be expressed in the olfactory epithelial cells, which can be used to activate the S protein to promote the infection and fusion of olfactory epithelial cells by coronaviruses. Moreover, the coronavirus can directly invade the CNS via the olfactory nerve. In the process, it transcribers and replicates, amplifying the intensity of infection and eventually destroying the brain tissue. Davies et al. (2020) believed that the NRP1 could mediate SARS-CoV-2 to enter the brain through the olfactory epithelium/olfactory bulb (olfactory bulb input to the olfactory nodules and then to the limbic system). In mouse models, researchers found that coronavirus could invade the CNS through this transcriptional pathway in the nasal cavity of mice. This view was also confirmed by intranasal inoculation of results of MERS-CoV and HCoV-OC43 mouse models (Zubair et al., 2020). So far, many clinical cases have proved that some patients had a loss of smell soon after infection with the coronavirus. When the disease was severe, the coronavirus could rapidly spread to several areas of the brain and brainstem, and eventually reach the spinal cord (Le Coupanec et al., 2015), with respiratory failure and other symptoms. As a result, nasal swabs and throat tests have become crucial tools to check whether the coronavirus has invaded the human body at a time, when the epidemic is becoming routine. People can use these tools to diagnose their diseases and take action.

#### 3.1.2. Receptor binding pathway

The receptor binding pathway is the approach through which virus particles contact and invade host cells by combining surface proteins with host cell receptors, thus infecting the human body. These seven human coronaviruses can affect the CNS by receptor binding pathway, but their receptors are not identical. Coronavirus receptors primarily include protein receptors and non-protein receptors. The aminopeptidase N, ACE2, and dipeptidyl peptidase 4 (also known as DPP4, a transmembrane receptor expressed on T cells) are the most common protein receptors. Also known as APN, the aminopeptidase N is a metal transmembrane glycoprotein of zinc ion-dependent membrane-bound type II and adhesive the receptor for all coronaviruses (Chen et al., 2016). SARS-CoV-2, SARS-CoV, and HCoV-NL63 use ACE2 as the receptors, while HCoV-OC43 and HCoV-HKU1 use 9-O-acetylated sialic acid as the receptors (Hulswit et al., 2019). What’s more, the HE protein encoded by HCoV-OC43 and HCoV-HKU1 also has acetylesterase activity, which can be used as a cofactor of S protein to promote the attachment of virions and mediate the enhancement of neurotoxicity (Paniz-Mondolfi et al., 2020) and P. MERS-COV and HCoV-229E use dipeptidyl peptidase 4 (DPP4) (Fehr and Perlman, 2015) and aminopeptidase (APN) (Hulswit et al., 2019) as receptors to infect macrophages, endocrine cells, ganglion dendritic cells, and astrocytes, respectively. Because some coronaviruses have the same receptors, they have overlapping routes of infection. For example, SARS-COV-2, SARS-CoV, and HCoV-NL63 can all infect the cells (e.g., glial cells, pericytes, epithelial cells, endothelial cells.) wrapped around cerebral blood vessels with the support of the receptor ACE2 (Alshebr et al., 2020), forming a temporary replication center. After the coronavirus is “localized” (the replication process stabilizes), it continues to infect and spread throughout the brain. Paniz-Mondolfi et al. (2020) found the presence of SARS-CoV-2 virus particles in the capillary endothelium and neurons of frontal lobe specimens, which confirmed the previous views. Besides, MERS-CoV invades T cells via the DPP4 receptor, which stimulates T cells to secrete lymphokines, enhances abnormal immune responses, induces cytokine imbalances, and ultimately leads to brain tissue inflammation. The development of ectopic antibodies to ACE2 is thought to induce a delayed immune response and further promote cytokine storm (Amiral et al., 2020), which may have similar effects to DPP4. Not only do individuals with MERS-CoV have this form of illness, but also do people with other coronaviruses. It can be associated with misdirected host immune responses in susceptible patients individuals (virus-induced neuro-immunopathology), which causes damage to the CNS cells (Desforges et al., 2019). The release of cytokines and chemokines in SARS-CoV-2 patients leads to immune-mediated neurological diseases, further supporting the above view (Zhao et al., 2020).

#### 3.1.3. Trojan horse mechanism

The virus infects mononuclear/macrophages through blood circulation, and the immune defense cells can naturally cross the BBB and move uncontrollably toward the brain tissue in a process called Trojan horse mechanism. Immune cells are constantly multiplied by the stimulation of the coronavirus, exerting an immune effect and destroying the structure and function of the NS. Also, the activation of coronavirus particles in the circulatory system enhances the release of inflammatory cytokines. This reaction can disrupt the integrity of the blood-brain barrier and affect its permeability, prompting T-cell invasion into the CNS, which can trigger inflammation of the brain tissue and further develop encephalitis (Ogier et al., 2020). Xu et al. (2005) detected neuronal necrosis and glial cell proliferation in patients with deceased encephalitis. In infiltrating monocytes and T cells, the researchers observed with electron microscopy that the viral particles in neuritis contained SARS-CoV RNA. McAbee et al. (2020) and Kim et al. (2017), respectively, found that a COVID-19 patient and a MERS patient both had encephalitis. Nilsson et al. (2020) demonstrated that the children with severe immunosuppression also had encephalitis, and the brain biopsies were found to be positive for HCoV-OC43 RNA by metagenome sequencing. Thus, it is speculated that an overactive immune response could also damage the nerve cells and affect brain function, causing the disorders such as cognitive impairment. Zhao et al. (2022) showed that the mild COVID-19 patients had a certain degree of cognitive impairment. Han and Song (2021) also confirmed that a patient with acute necrotizing encephalitis caused by HCoV-NL63 infection had severe cognitive impairment.

#### 3.1.4. Neurons retrograde pathway

**FIGURE 1:**
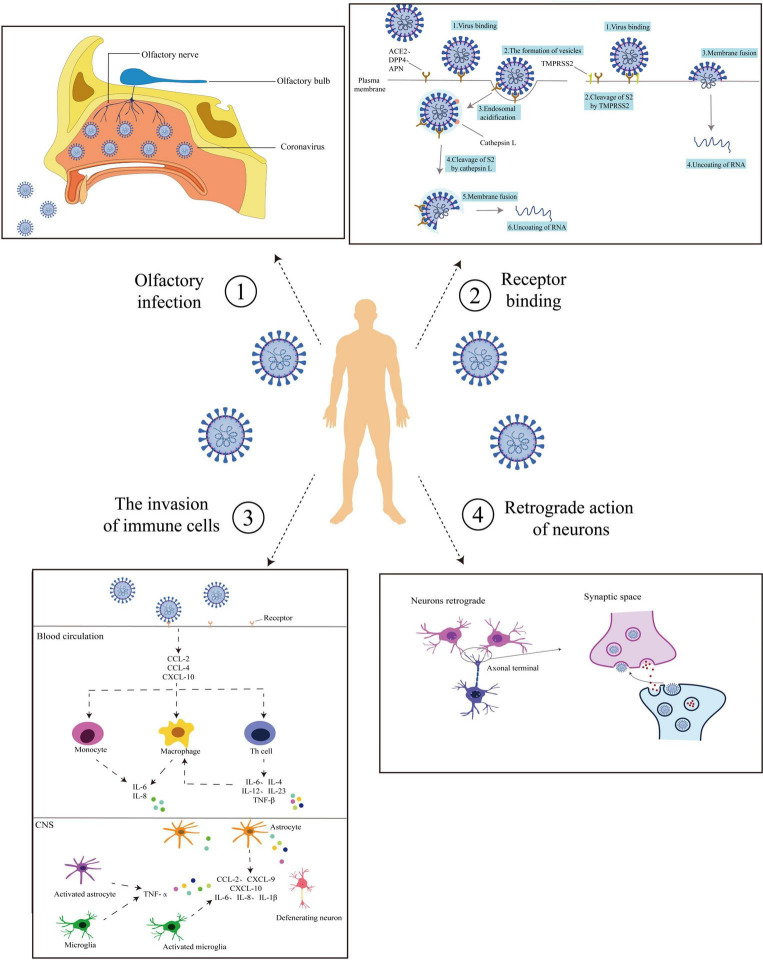
Routes of coronaviruses infection. Coronavirus entry routes into the central nervous system (CNS). ➀ Olfactory pathway: Coronaviruses enter from the mouth and bind to the surface receptors of the olfactory nerve. It is transmitted by the olfactory nerve to the olfactory bulb and thence to the nerve center. ➁ Receptor binding: Coronaviruses bind to the host cell receptors by the surface proteins with the assistance of TMPRSS2. On the one hand, coronaviruses enter the host cells through endocytosis and use Cat L to clear S2. Thus coronaviruses can use plasma membrane fusion so that nucleic acid is released. On the other hand, coronaviruses fuse with the host cell membrane through the viral outer membrane, allowing the nucleic acid to enter the host cell. ➂ Trojan horse mechanism: Coronavirus-mediated cytokine storm. After attachment and entry into the epithelial cells through the ACE-2 receptor, the virus may activate the pro-inflammatory pathway through NF-κB signaling, followed by the formation of an inflammasome. Various pro-inflammatory cytokines and chemokines are released, including CCL-2, CCL-4, and CXCL-10. These proteins attract different immune cells in the circulation, like the monocytes, macrophages, and T cells at the site of infection. Additionally, the T lymphocytes release substances (e.g., TNF-β, IL-6, IL-4, IL-12, and IL-23) that in turn affect macrophages and accumulate in the body, creating a pro-inflammatory feedback loop. These cytokines may damage the BBB and activate astrocytes and microglia. In response, the activated microglia and astrocytes produce IL-1β, IL-6, TNF-α, and IL-8. Elevated levels of these inflammatory cytokines can impart neurotoxic effects leading. ➃ Neurons retrograde: After infection of peripheral neurons, coronaviruses spread through retro-transsynaptic neurons (from postsynaptic neurons to presynaptic neurons in reverse), and infect synaptic connected central neurons or other peripheral neuron cell bodies. The solid line shows the process of virus infection, and the dashed line shows the possible route of virus infection.

After infection of peripheral neurons, coronaviruses can infect synaptic neurons connected to central neurons or other peripheral neuron cell bodies by retrograde transsynaptic neuron propagation (reverse movement from postsynaptic neuron to presynaptic neuron) (Rabaan et al., 2020). HCoV-OC43 destroyed the CNS through the mechanism of retrograde rapid axonal transport that infected neuronal cell bodies (Dubé et al., 2018). Wei et al. (2018) observed that SARS-CoV would pass through the olfactory bulb (the first-level processing station of the olfactory system, mainly composed of the mitral cells, axonal fibers of tufted cells, and fibers projected from the olfactory cortex to the olfactory bulb) in the mouse model. This study found that coronavirus could enter the globular olfactory layer utilizing olfactory neuron axons, and form synaptic connections with the mitral cells and plexiform cell dendrites in the olfactory bulb. Netland et al. (2008) indicated that when the coronavirus entered the central nervous system, it would exhibit rapid transsynaptic diffusion, which provided a basis for it to invade the human central nervous system. Moreover, some coronaviruses appear to be able to transport using a signal that is commonly involved in retrograde transport pathways (neurotrophic factors). In this signaling pathway, neurotrophic factors and their receptors are incorporated into vesicles containing the coronavirus. When the virus matures, neurotrophic factors promote reverse vesicle transport (Koyuncu et al., 2013). The mechanism provides a reference for the substances involved in the retrograde transport of coronavirus in neurons. Retrograde transport can use the way of endocytosis and exocytosis to fuse with the cell membrane of host cells and form vesicles for reverse transport, thus leading to central nervous system diseases. Paniz-Mondolfi et al. (2020) found that SARS-CoV-2 particles were wrapped in the expanded vesicles. The results of electron microscopy showed that SARS-CoV-2 particles underwent endocytosis or exocytosis in endothelial cells, which was consistent with the above views. The four coronavirus infection pathways, as described above, are now summarized in Figure 1.

The above content describes the main ways and mechanisms of the human CNS damaged by coronaviruses, which helps people to understand the invasions of coronaviruses more accurately. The types, names, and routes of entry into the CNS of various coronaviruses are summarized in Table 1.

**TABLE 1:** Classification, properties, cell receptors, and routes of coronaviruses that infect humans into the CNS.

Genome	Virus family	N/E	Viruses	Cell receptor	CNS entry
ssRNA	Coronavirus family	E	SARS-CoV	ACE2	Olfactory system, Receptor binding, Trojan horse mechanism, Neurons retrograde
			SARS-CoV-2	ACE2	Olfactory system, Receptor binding, Trojan horse mechanism, Neurons retrograde
			MERS-CoV	DPP4	Olfactory system, Receptor binding, Trojan horse mechanism
			HCoV-OC43	*N*-Acetyl-9-0-acetylneuraminic acid (9-0-acetylsialic acid)	Olfactory system, Receptor binding, Trojan horse mechanism, Neurons retrograde
			HCoV-HKU1	*N*-Acetyl-9-0-acetylneuraminic acid (9-0-acetylsialic acid)	Receptor binding
			HCoV-229E	APN	Receptor binding
			HCoV-NL63	ACE2	Receptor binding, Trojan horse mechanism

ssRNA, single-stranded RNA; N, naked; E, enveloped.

### 3.2. Mechanisms of the PNS injury caused by coronaviruses

The PNS is composed of cranial and spinal nerves. In terms of function, the PNS can be divided into sensory nerves and motor nerves, which have different functions due to the different distribution locations of neurons. The peripheral protrusions of sensory neurons are distributed throughout the body along with the spinal nerves, and their endings form various receptors that sense various stimuli. Meantime, motor neurons can be found in skeletal muscle, heart muscle, smooth muscle, and glands. These neurons primarily control the human body’s movement and the voluntary movement of internal organs, such as the movement of the skeletal muscles and the peristalsis of the intestinal tract. According to the state of coronavirus invasion of the PNS, the ways of invading peripheral neurons can be classified into two pathways: direct destruction and indirect destruction.

#### 3.2.1. Direct destruction of the peripheral neurons

Neuromuscular symptoms were reported in patients with SARS-CoV from Taiwan, China (Tsai et al., 2004). This symptom was mainly consistent with severe polyneuropathy (CIP) or myopathy with elevated creatine kinase. It was previously thought that the levels of creatine kinase in SARS-CoV patients were an indicator of muscle damage or decay. However, the study later refuted this viewpoint, emphasizing that even patients with no change in creatine kinase levels had necrosis or atrophy of muscle fibers (Tsai et al., 2004). This suggested that SARS-CoV could indeed have a significant effect on the PNS, especially at the neuromuscular junction. Symptoms of the PNS infection with coronaviruses (such as SARS-CoV-2) include dyskinesia, hypogeusia, and certain diseases. For example, Guillain-Barre syndrome (GBS) is an autoimmune peripheral neuropathy characterized by demyelinating lesions of peripheral nerves and nerve roots and infiltration of inflammatory cells in small vessels. Turgay et al. (2015) found that a Turkish patient was conscious, but swallowing, chewing, and speech functions were damaged, and his deep tendon reflex was lost. Real-time polymerase chain reaction (PCR) analysis of nasal swab samples was reported to be positive for HCoV-229E and HCoV-OC43, and HCoV co-infection was diagnosed. A child patient in Saudi Arabia (Lee et al., 2003) presented with GBS, which was characterized by leg weakness, loss of deep tendon reflexes, tingling in the lower limbs, and proprioceptive disturbance. The patient’s nasopharyngeal swab was SARS-CoV-2 positive 22 days after symptom onset. Based on the above, it can be concluded that the invasion of coronavirus can destroy nerve endings, which cannot transmit nerve impulses, resulting in the abnormal work of the nerve-muscle junction. Patients may suffer from muscle weakness and loss of muscle reflexes. Martinez et al. (2020) found that SARS-CoV-2 could enter the brain through the glossopharyngeal nerve or directly damage the glossopharyngeal nerve, affecting the formation of taste. A study (Mao et al., 2020) analyzed the symptoms of COVID-19 patients in three central districts of Wuhan, China, and found that 12 out of 214 patients (5.6%) had impaired taste. Although the proportion is small, it is still not negligible.

#### 3.2.2. Indirect destruction of the peripheral neurons

Vonck et al. (2020) found that some COVID-19 patients showed symptoms of GBS due to autoimmune and inflammatory syndrome. Ozonoff et al. (2021) observed that the incidence of Bell’s palsy (facial neuritis) in the Pfizer vaccinated population was 3.5–7 times higher than would be expected in the general population. It has been speculated that this vaccine-induced innate immune activation through the combined action of mRNA and lipids to produce interferon. Interferon can affect the synthesis of coronavirus proteins by acting on receptors in uninfected cell membranes, but it also increases the lethality of T cells and may temporarily disrupt the peripheral tolerance, thereby interfering with peripheral nerve function (Ma et al., 2019). Various substances and interferon brought by inflammation may be essential factors in the indirect destruction of the PNS by coronaviruses (Yalçindaǧ and Alay, 2013).

This view provides a new idea for the treatment and research of peripheral nerve diseases caused by coronavirus infections in the future, and can be more accurately understood through the clinical manifestations of patients.

## 4. Discussion

### 4.1. Relationship between structural proteins and virulence of coronaviruses

The ability of coronaviruses to infect NS is related to structural proteins on the envelope, such as S proteins and HE proteins, which can bind to receptors on the surface of host cells. And the virulence of coronaviruses carrying these proteins changes as their activity varies. In short, the stronger the protein activity, the more virulent the coronavirus. Because the affinity between SARS-CoV-2 S protein and ACE2 is higher than that of SARS-CoV, the former has a higher ability to infect host cells than the latter. Especially in the “receptor binding pathway” in which coronavirus invades the CNS, as the “right-hand man” of coronavirus, the S protein can constantly bind new receptors and destroy host cells with the spread of coronavirus. Additionally, it is crucial to recognize the role that the HE protein plays. It can be used as both a mediator and a cofactor to bind receptors, and can also promote the binding of S protein to receptors, speeding up the rate of virus invasion. Furthermore, the E protein induces an immune response that “accidentally wounds” the normal cells, causing them to be damaged or die. The N protein can participate in and promote viral replication, and accelerate the adaptability of the virus to the host. As a result, several drug research and development teams are attempting to stop the proliferation and pathogenicity of coronaviruses by destroying the glycoproteins from their surface. At the same time, these proteins have become the markers for identifying coronaviruses. It can be considered whether the specific recognition of immune cells promotes the occurrence of human-specific immunity, and finally eliminates the coronaviruses. It is also a crucial idea for vaccine research.

### 4.2. Comparison of the effects of coronaviruses on the CNS and the PNS

After entering the body, coronaviruses can rely on unique structural proteins to invade host cells (e.g., neurons, glial cells.) and affect the NS function. The mechanisms of coronavirus invasion into the CNS and the PNS are comprehensively described in this article. The patient’s performance varies as a result of the coronavirus infecting the two nervous systems. Thereby, the infection routes and clinical manifestations of the CNS and the PNS caused by coronaviruses are analyzed and compared, as shown in Table 2.

**TABLE 2:** Comparison of infection routes and clinical manifestations of the CNS and the PNS caused by coronaviruses.

Type	CNS				PNS	
Route of infection	Neurons retrograde	Receptor binding	Olfactory system	Trojan horse mechanism	Direct destruction	Indirect destruction
Clinical manifestation	Headache, dizziness, cognitive impairment, acute cerebrovascular disease, epilepsy.		Reduced or even loss of smell and related neurological disorders.	Encephalitis, neuritis, and other inflammatory diseases of the nervous system.	Symptoms, Muscle atrophy, muscle weakness, loss of muscle reflexes, inability to move freely, and hypogeusia, neuralgia.	Guillain-Barre syndrome (GBS), facial and neuritis.

As shown in Table 2, there is a specific correlation between the routes of coronavirus infection and clinical manifestations. If the coronavirus infects mononuclear/macrophage, it will induce various inflammatory reactions in the nervous system. Damage to the olfactory nerve in the olfactory system can lead to hyposmia. The destruction of neurons by the above pathways will prompt patients to show related neurological symptoms. Furthermore, there is a close relationship between the CNS and the PNS. Coronaviruses can not only invade the CNS through the PNS, such as the olfactory nerve. At the same time, it can also affect the PNS from the CNS in turn. For example, the damaged corticospinal tract will lead to the loss of voluntary activities of the human body. Hence, in treating nervous system diseases caused by a coronavirus, it is necessary to pay attention to which nerve tissue cells are directly connected to the infected site. This can reduce the adverse chain reaction caused by treatment and reduce the risk of treatment.

### 4.3. The connection between the nervous system transmission of coronaviruses and its therapeutic agents

For the combination of coronaviruses (such as SARS-CoV-2) and the olfactory epithelial cells in the nasopharynx, humans can prevent the invasion of coronaviruses on the epithelial mucosa and olfactory epithelial cells by covering the nose and mouth. With this measure, humans can effectively prevent the coronavirus from entering the blood circulation and prevent a range of respiratory and nervous system diseases. If a person is already infected with such a virus, he or she will need to take the medication as prescribed by his or her doctor. The action mechanisms of drugs usually prevent coronaviruses from binding to ACE2 receptors on host cells, by destroying the structure of viral proteins or binding to viral proteins. The result is to reduce the infectivity of coronaviruses to host cells and achieve the therapeutic effect. Currently, it is known that the antibiotic ceftazidime could bind to the RBD domain of S protein and inhibit the binding of S-RBD to ACE2, thus blocking the invasion of coronaviruses (Lin et al., 2021). Another experiment (Hoffmann et al., 2020c) showed that chloroquine/hydroxychloroquine also had an antiviral effect. Cathepsin L (Cat L) is a class of proteolytic enzymes that can cleave peptide bonds and handle SARS-CoV-2 S protein. This enzyme is present in the kidney cells (experimental cells) of African green monkeys, and its function is affected by cell pH. Coincidentally, chloroquine/hydroxychloroquine can indirectly inhibit the invasion of SARS-CoV-2 by regulating pH to restrict the function of the Cat L enzyme (Hoffmann et al., 2020c). Besides, due to the Trojan horse mechanism, the body will trigger a robust autoimmune response, which damages neurons and tissue cells, forming encephalitis. The condition can also be treated with chloroquine phosphate, which effectively inhibits interferon and Interleukin-6 (IL-6), preventing further damage to the brain cells. On the contrary, another study (Hoffmann et al., 2020a) held a different view, holding that there were specific differences between experimental cells and susceptible cells of the human body, which should not be generalized. More effectively, the promotion effect of some enzymes (such as TMPRSS2) on coronavirus protein was utilized, and the emphasis was placed on reducing the infectivity of coronaviruses by inhibiting the production of this enzyme. So, protein inhibitors have been proposed as therapeutic drugs for coronaviruses, such as TMPRSS2 inhibitors. Moreover, after infecting the host cell, coronaviruses used the energy and material of the host cell for transcription, replication, and translation, thus expressing their genetic material. This result led to damage to the physiological structure, affecting life activities and causing death in severe cases. In response, researchers have identified inhibitors of the enzymes that replicated the SARS-CoV-2 gene – Masitinib (Drayman et al., 2021) and Remdesivir (Uzunova et al., 2020). These two drugs can block the replication of SARS-CoV-2, reduce the level of inflammatory cytokines and decrease the inflammatory response, which can be used in the research and development of SARS-CoV-2 drugs. These drugs also provide research direction for treating SARS-CoV, MERS-CoV, HCoV-OC43, and other coronaviruses, and can significantly inhibit various diseases caused by coronaviruses.

**FIGURE 2:**
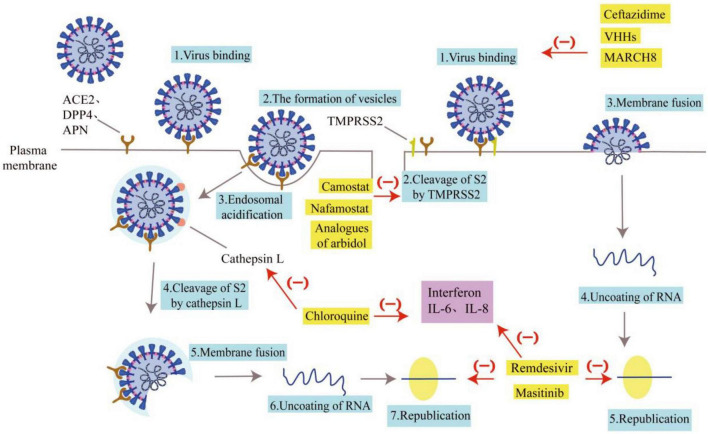
Pathways of drug inhibition. Some drugs are listed for their role in inhibiting the entry of coronaviruses. The gray arrows represent the invasion route and replication process of the coronavirus, and the blue boxes represent the name of the invasion link. The red arrows and “(–)” indicate the drug’s resistance to coronavirus invasion, and the yellow boxes indicate the name of the drug. Ceftazidime, VHHs, and MARCH 8 can inhibit the binding of coronaviruses to receptors. Analogs of arbidol, Camostat, and nafamostat can impact how well TMPRSS2 clears S2. Chloroquine can prevent Cat L from clearing S2. The coronavirus nucleic acids can’t replicate when treated with Remdesivir and Masitinib. The purple boxes show interleukin and interferon secreted by immune cells. Interferon, IL-6, and IL-8 actions can be inhibited by chloroquine, Remdesivir, and Masitinib, which will lessen the inflammatory response.

According to the inhibitory effects of drugs on coronaviruses, the inhibitory pathways of drugs can be plotted, as shown in Figure 2.

### 4.4. Comparison of infectivity and mortality of seven coronaviruses

The infectivity of a virus is determined by both the route of transmission and the intensity of infection. Among the seven human coronaviruses, SARS-CoV-2, SARS-CoV, MERS-CoV, and HCoV-OC43 have the most transmission paths, so they are more obvious in patients with more diverse symptoms. Nonetheless, unlike the high fatality rates of viruses such as SARS-CoV and MERS-CoV, the mortality rates of patients infected with SARS-COV-2 and HCoV-OC43 are not high. This means that the transmission path is not directly related to the fatality rate, which is more due to the infectivity of the coronavirus and the patient’s pre-existing illness. SARS-CoV-2, for example, worsens or kills patients mainly by inducing or aggravating their existing diseases, so the fatality rate among middle-aged and elderly patients is much higher than among teenagers. In contrast, the remaining common coronaviruses (e.g., HCoV-229E, HCoV-NL63, and HCoV-HKU1) are less infectious because of their limited transmission routes and infectivity, and the diseases they cause are mostly mild and usually present with symptoms associated with respiratory tract infections.

## 5. Summary

This article reviews the mechanisms of coronavirus infection in the NS, and thoroughly explains the impacts of the viruses on the PNS and the CNS, which can effectively help people to prevent the infection of the coronavirus, reduce the damage of the coronavirus to the human body, and contribute to the prevention of the infection of the coronavirus. Further, according to the NS invasion mechanisms of different coronaviruses, the consequences and symptoms of individuals infected with the coronavirus may be assessed, and all diseases brought on by coronaviruses can be purposefully treated. Therefore, the exploration of the mechanisms of coronavirus invasion into the NS can provide a theoretical basis for the research and development of related drugs, further promote the prevention and treatment of coronavirus infectious diseases, and contribute to the global epidemic prevention cause.

## Author contributions

AS conceived and planned the overall structure of the review, wrote the manuscript, and approved the final manuscript. HC collected the references and wrote the manuscript. AS and HC collaboratively finished the revision of the manuscript. Both authors contributed to the article and approved the submitted version.
